# Construction and validation of a prognostic risk model of angiogenesis factors in skin cutaneous melanoma

**DOI:** 10.18632/aging.203895

**Published:** 2022-02-14

**Authors:** Songyun Zou, Yonggang Zhang, Limei Zhang, Dengchuan Wang, Shi Xu

**Affiliations:** 1 Department of Burn and Plastic Surgery, Shenzhen Longhua District Central Hospital, Shenzhen, Guangdong, China; 2 Department of Clinical Laboratory, Shenzhen Longhua District Central Hospital, Shenzhen, Guangdong, China; 3 Oncology Department, Shenzhen Longhua District Central Hospital, Shenzhen, Guangdong, China; 4 Office of Medical Ethics, Shenzhen Longhua District Central Hospital, Shenzhen, Guangdong, China

**Keywords:** skin cutaneous melanoma, angiogenesis factors, prognostic risk model, gene signature, risk score, immune

## Abstract

Melanoma can secrete tumor angiogenesis factors, which is the essential factor for tumor growth and metastasis. However, there are few reports on the relationship between angiogenesis factors and prognosis risk in melanoma. This study aimed to develop a prognostic risk model of angiogenesis for melanoma. Forty-nine differentially expressed angiogenesis were identified from the TCGA database, which were mainly involved in PI3K/Akt pathway, focal adhesion, and MAPK signaling pathway. We then establish an eleven-gene signature. The model indicated a strong prognostic capability in both the discovery cohort and the validation cohort. Patients of smaller height (<170 cm) and lower weight (<80 kg) and those with advanced-stage and ulcerated melanoma had higher risk scores. The risk score was positively correlated with mutation load, homologous recombination defect, neoantigen load and chromosome instability. In addition, the high-risk group had a higher degree of immune cell infiltration, better response to immunotherapy and lower immune score. Therefore, these results indicate that the risk model is an effective method to predict the prognosis of melanoma.

## INTRODUCTION

Skin cutaneous melanoma (SKCM) is a malignant skin tumor arising from the malignant transformation of melanocytes [1]. Although melanoma is less common than other skin cancers, it is more lethal, accounting for approximately 73% of skin cancer-related deaths [2]. According to the report from International Agency for Research on Cancer (IARC), it is estimated that there are more than 280,000 new cases and more than 60,000 related deaths each year worldwide [3]. The incidence and mortality rate are significantly different in different countries, mainly due to the timing of diagnosis and treatment [4]. The 5-year survival rate of patients with cutaneous melanoma at stage 0 is 97%, while the relative survival rate of patients with cutaneous melanoma at stage IV is only approximately 10% [5]. Melanoma is caused by interactions between genetic susceptibility and environmental exposure [4]. Although melanoma usually occurs on the skin, it may also migrate to other areas of the body, including the eye, gastrointestinal tract, urogenital system and nasopharynx with neural crest cell involvement [1, 5]. Metastasis is also the main cause of death in patients with melanoma [6]. Early diagnosis of malignant skin cutaneous melanoma is difficult, and the prognosis is poor. Although some risk factors are known, early diagnosis and treatment are still the only strategies to improve prognosis [7]. Therefore, it is critical to establish a multidimensional model to characterize the processing of melanoma.

Solid tumors can secrete angiogenesis factors (AFs), which can induce angiogenesis and promote tumor growth. AFs are significantly correlated with tumor invasion and metastasis, tumor stage and the survival rate of patients with melanoma, colorectal cancer, pancreatic cancer and other tumors [8–11]. AFs served as important targets in treating of melanoma and other tumors [12, 13], however, to the best of our knowledge, there still no related prognostic risk model been reported. This study aimed to develop a prognostic risk model of AFs and a nomogram for melanoma, which herein investigate the association of the risk score with clinical features, genetic characteristics, mutations and immune landscapes.

## RESULTS

### Identification of DE-AFs

We obtained 470 tumor and 737 normal tissues after excluding patients with incomplete data. GSE65904 [14] was used as the validation dataset, including 214 melanoma items and the corresponding survival data. The sample statistics are shown in Table 1 (more detailed clinical characteristics of melanoma patients are shown in Supplementary Tables 1 and 2).

**Table 1:** Statistical table of clinical information.

	**TCGA-SKCM**	**GSE65904**
Alive	247	108
Dead	223	106
Age (> = 60 years)	221	133
Age (<60 years)	241	81
Survival Time (>365 days)	398	135
Survival Time (<365 days)	72	75

**Figure 1:**
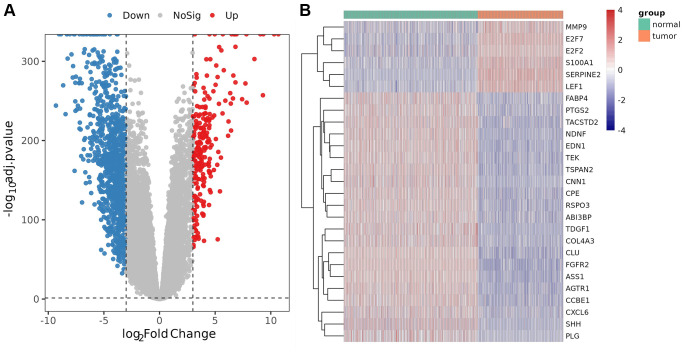
**Analysis of differentially expressed genes (DEGs) and DE-AFs.** (**A**) Volcano plot of DEGs between tumor and normal samples. (**B**) Heatmap of DE-AF expression between tumor and normal samples. Tumor samples and normal samples are shown in green and orange, respectively. Red indicates genes that had higher expression levels, and blue indicates genes with lower expression levels.

Compared with the control, a total of 2152 DEGs (Supplementary Table 3) were identified in melanoma samples from the TCGA dataset, which are shown in the volcano map (Figure 1A). Forty-nine DE-AFs were obtained by overlapping the AF gene set and DEGs, consisting of 26 upregulated and 23 downregulated DE-AFs. There were significant differences in the expression levels of the 49 DE-AFs between tumor samples and normal samples through hierarchical cluster analysis, as shown in Figure 1B and Supplementary Table 4.

### KEGG enrichment analysis for the DE-AFs

**Figure 2:**
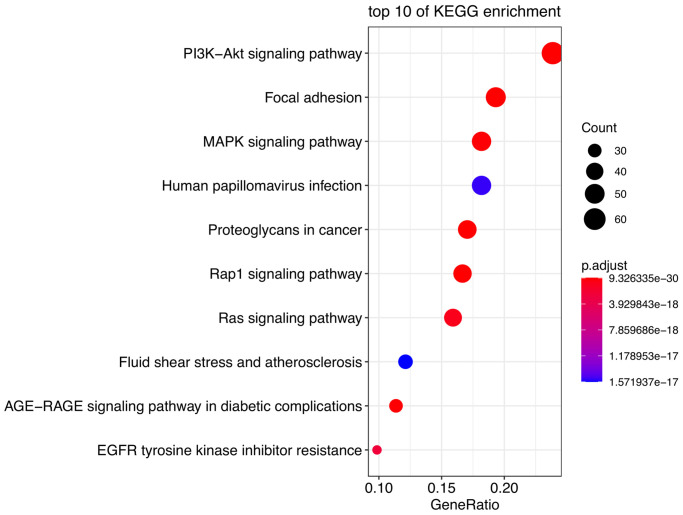
**Pathway enrichment analysis of DE-AFs.** Kyoto Encyclopedia of Genes and Genomes (KEGG) pathway enrichment analysis of DE-AFs.

The most significant KEGG pathways were analyzed to reveal the potential biological functions of the DE-AFs. As shown in Figure 2, the DE-AFs were mainly involved in PI3K/Akt pathway, focal adhesion and MAPK signaling pathway. The most significant GO terms were also analyzed to reveal the potential biological functions of the DE-AFs (Supplementary Figure 1).

### Construction and validation of the prognostic gene signature based on DE-AFs

**Figure 3:**
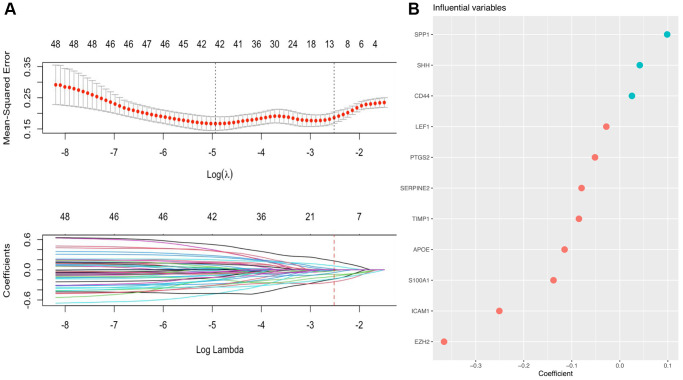
**A prognostic gene signature based on AFs was established by LASSO regression analysis.** (**A**) Determination of the number of factors by LASSO analysis. The mean square error distribution and the coefficient distribution of all variables under different lambda are from top to bottom. (**B**) The distribution of significant coefficient variables.

**Figure 4:**
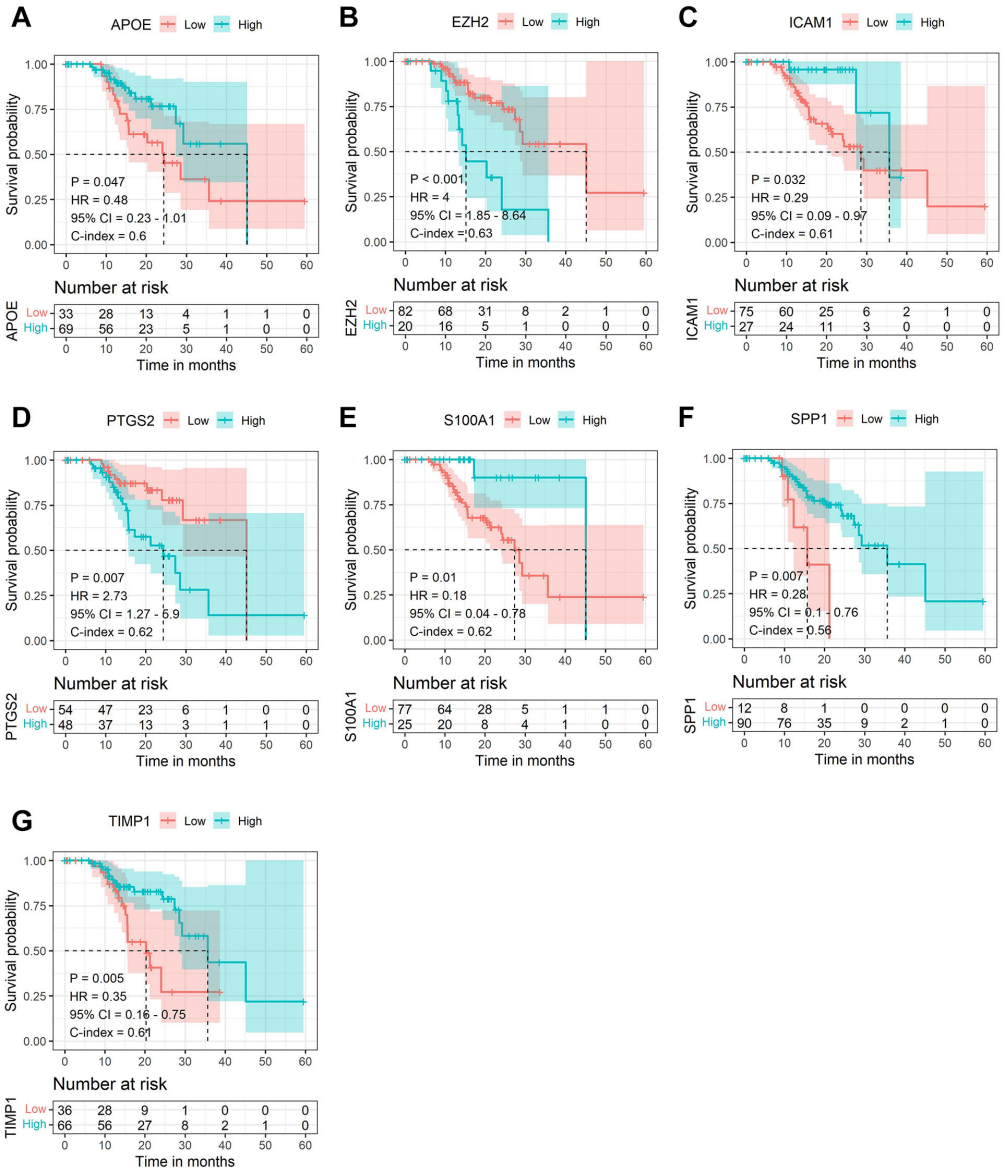
**The Kaplan-Meier survival analysis of the selected DE-AFs.** Survival analysis of the selected DE-AFs in the TCGA cohort, APOE (**A**), EZH2 (**B**), ICAM1 (**C**), PTGS2 (**D**), S100A1 (**E**), SPP1 (**F**) and TIMP1 (**G**). High and low expression is shown in blue and red, respectively.

Subsequently, LASSO Cox regression analysis was performed to further analyze these DE-AFs, and the significant features were visualized by sorting the coefficients (Figure 3). Next, we built an eleven-gene signature, and the risk score was defined as follow formula: risk score = −0.12 × APOE + 0.03 × CD44 − 0.37 × EZH2 − 0.25 × ICAM1 − 0.03 × LEF1 − 0.05 × PTGS2 − 0.14 × S100A1 − 0.08 × SERPINE2 + 0.04 × SHH + 0.1 × SPP1 − 0.08 × TIMP1. To evaluate the prognostic values of all selected DE-AFs, survival curves for melanoma patients were plotted. The overexpression of the selected 7 DE-AFs was significantly and negatively associated with the prognosis of melanoma patients (Figure 4A–4G).

**Figure 5:**
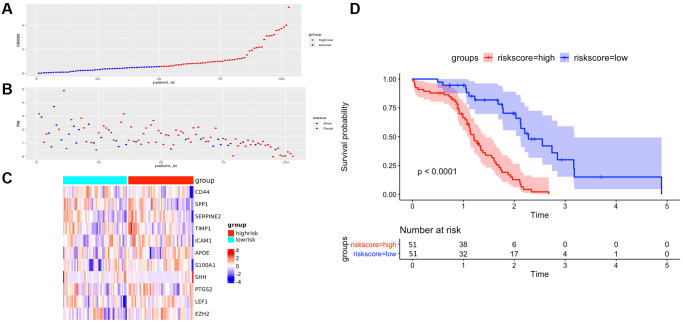
**The performance of overall survival (OS) in the TCGA cohort based on the 11-AF signature.** (**A**) The distribution of high-risk and low-risk patients based on the risk score ranking. (**B**) The survival duration and status of the patients. The horizontal axis is the sample, and the vertical axis is the survival time. (**C**) Heatmap of significant survival-related DE-AFs between high- and low-risk patients. (**D**) Kaplan–Meier survival curves of overall survival of the high- and low-risk groups stratified by the risk score calculated by the 11-AF signature risk prediction formula.

**Figure 6:**
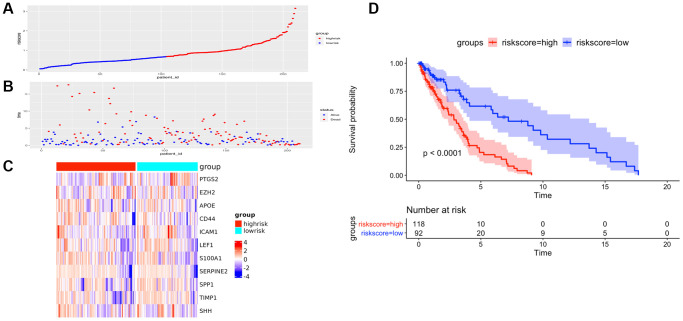
**The prognostic performance of the AF signature risk score in the validation set.** (**A**) The distribution of high-risk and low-risk patients based on the risk score ranking. (**B**) The survival duration and status of the patients. The horizontal axis is the sample, and the vertical axis is the survival time. (**C**) Heatmap of significant survival-related DE-AFs between high-risk and low-risk patients. (**D**) Kaplan–Meier survival curves of overall survival of the high- and low-risk groups stratified by the risk score calculated by the 11-AF signature risk prediction formula.

**Figure 7:**
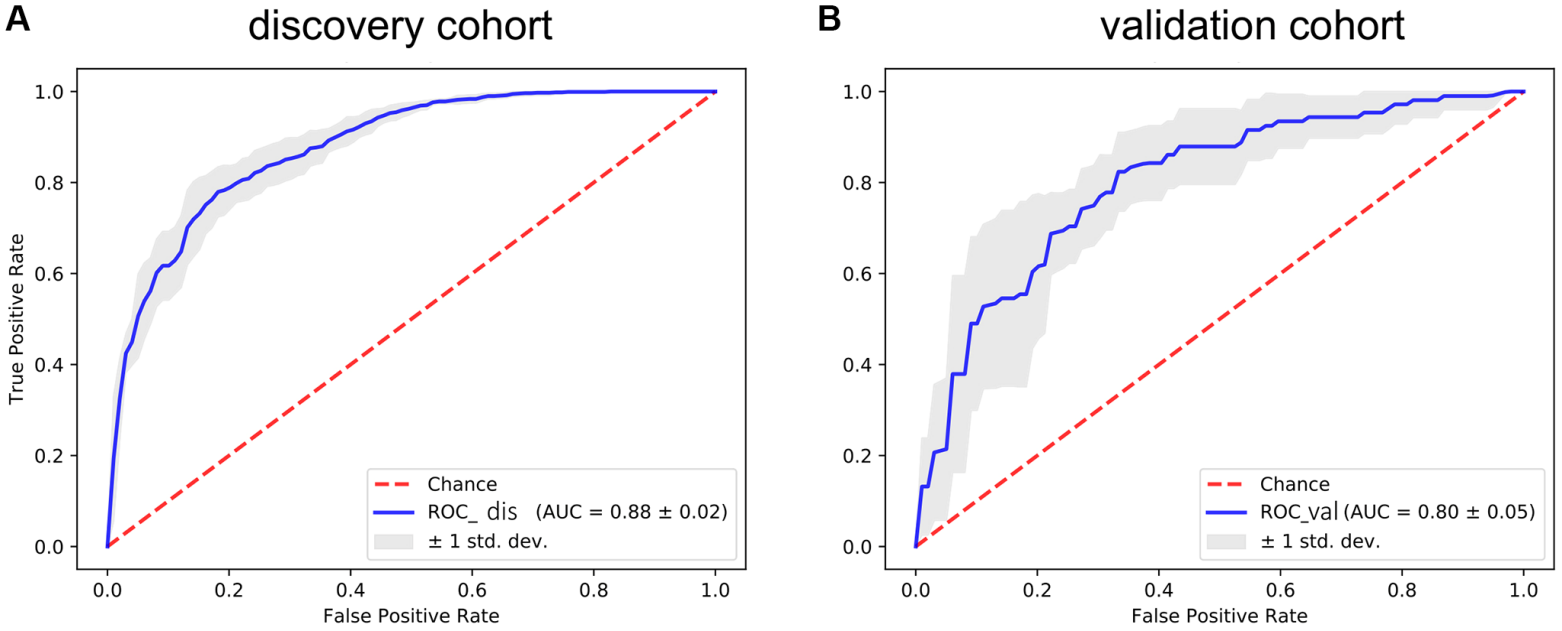
**The ROC curve for assessing the predictive power of the AF signature in the discovery cohort and validation cohort.** (**A**) The ROC curve of the discovery cohort. (**B**) The ROC curve of the validation cohort. The red dotted line is the random line, the blue curve is the AUC curve, and the gray line is the confidence interval.

To further verify the efficacy of this model, all patients were divided into low-risk group and high-risk group based on the median value of the risk score in the TCGA dataset (Figure 5) and the GEO verification dataset (Figure 6). Compared with the low-risk group, the patients in the high-risk group had poorer prognoses. Meanwhile, AUCs (Figure 7) were applied to estimate the predictive power of these prognostic models. The AUC values showed greater than 0.8 in both cohorts, which indicates that the model has high accuracy and stability.

### The association between the risk score and clinical and genetic characteristics

**Figure 8:**
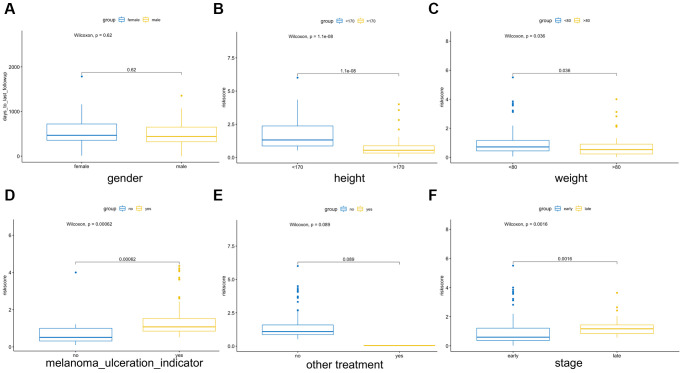
**Association between the AF signature risk score and clinical features.** These features included sex (**A**), height (**B**), weight (**C**), melanoma ulcer (**D**), other treatments (**E**) and clinical stage (**F**). Blue and yellow correspond to different groups of samples.

**Figure 9:**
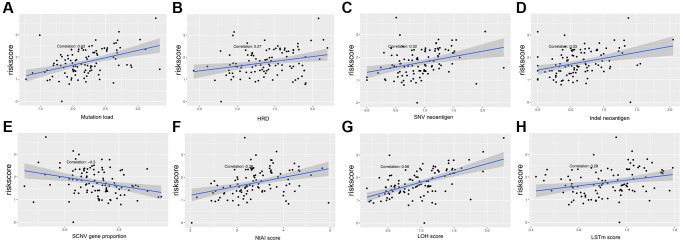
**Association between the AF signature risk score and genetic characteristics.** (**A**–**H**) The axis in the figure corresponds to mutation load, HRD, SNV neoantigen level, indel neoantigen level, SCNV gene proportion, NtAI score, LOH score and LSTm score. The vertical axis is the risk score, and the red line is the fitted regression line.

Multiple clinical features were collected, and respectively classified into two groups based on the median risk score. We calculated the significance value with the Wilcoxon test, the results are shown in Figure 8. Patients with smaller height (<170 cm) and lower weight (<80 kg) and those with advanced stage and ulcerated melanoma had higher risk scores. Moreover, other genetic characteristics, such as tumor mutation burden (TMB), homologous recombination defect (HRD), neoantigen load and chromosomal instability, were used to conduct a correlation analysis with the risk score. As shown in Figure 9, the risk score was positively correlated with mutation load, homologous recombination defect (HRD), neoantigen load (SNV- and indel-neoantigen) and chromosome instability (NtAI, LOH and LSTm score), while was negatively correlated with somatic copy number variations (SCNV) gene proportion.

### Immunocyte infiltration, immune score, and immunotherapy predictive efficiency

**Figure 10:**
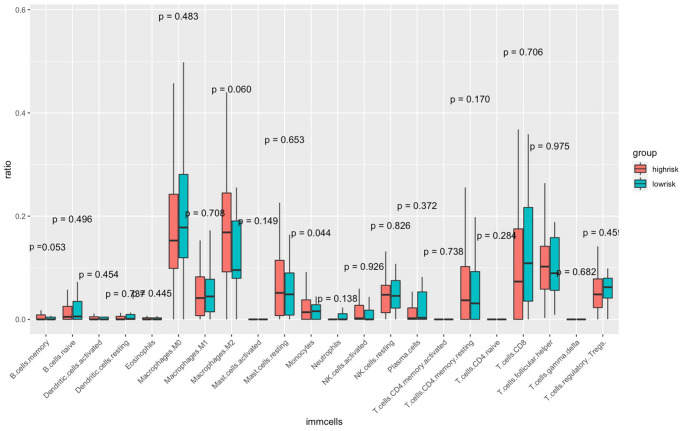
**Comparisons of the abundance of infiltrating immune cells between the high- and low-risk groups.** The horizontal axis is the immune cells, and the vertical axis is the infiltration fraction. High-risk and low-risk patients are marked in red and blue, respectively.

**Figure 11:**
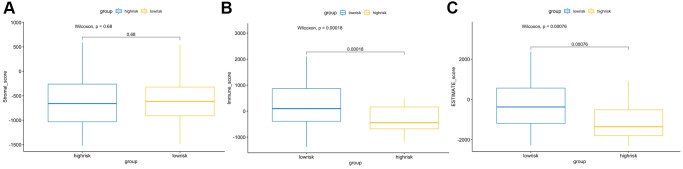
**ESTIMATE algorithm analysis of different risk score groups.** Comparisons of stromal (**A**), immune (**B**) and ESTIMATE (**C**) scores between the high- and low-risk groups.

**Figure 12:**
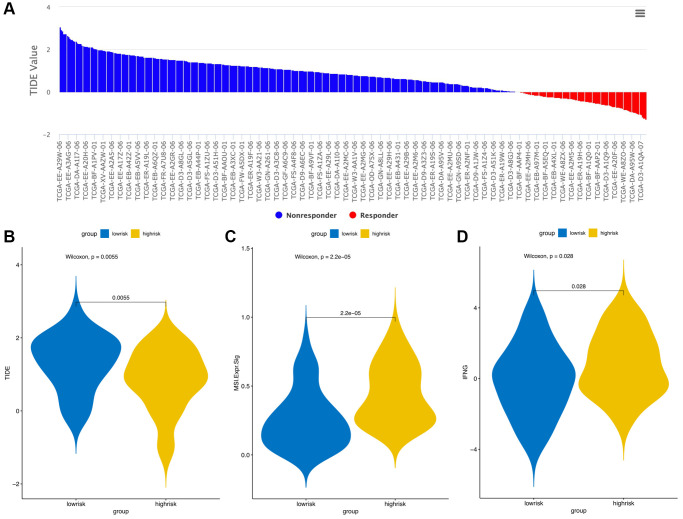
**TIDE signatures predict immunotherapy response based on risk score.** A waterfall plot of TIDE prediction scores in the TCGA cohort (**A**). Comparisons of TIDE (**B**), MSI (**C**) and IFGN (**D**) prediction scores between the high- and low-risk groups.

The CIBERSORT algorithm was used to quantitatively evaluate the infiltration degree of 22 types of immune cells in each patient, and the Wilcoxon test was used to calculate the difference in the level of each immune cell among patients with different risk scores. Compared with the low-risk group, the high-risk group had a higher level of infiltration of memory B cells, macrophages and monocytes (Figure 10). The Estimation of Stromal and Immune cells in Malignant Tumor tissues using Expression data (ESTIMATE) database is a tool to predict tumor purity and infiltrating stromal/immune cells in tumor tissues by using gene expression data. The proportions of stromal cells and immune cells in tumor samples and infer the purity of tumor tissues were further calculated [15]. We subsequently evaluated the differences in immune score, stromal score and ESTIMATE score between the high- and low-risk groups, the results are shown in Figure 11. These results show that there are significant differences between the immune score and tumor purity among high- and low-risk samples, and the patients with low-risk scores have higher immune scores. Moreover, as shown in Figure 12, the response to PD-1/CTLA4 immunotherapy of each sample was predicted by using the TIDE algorithm. There were significant differences in TIDE score, microsatellite instability and IFGN signature expression between the high- and low-risk groups. The high-risk group had higher microsatellite stability and IFGN signature expression and a lower TIDE score.

### Mutation patterns and copy number variant (CNV) analyses

**Figure 13:**
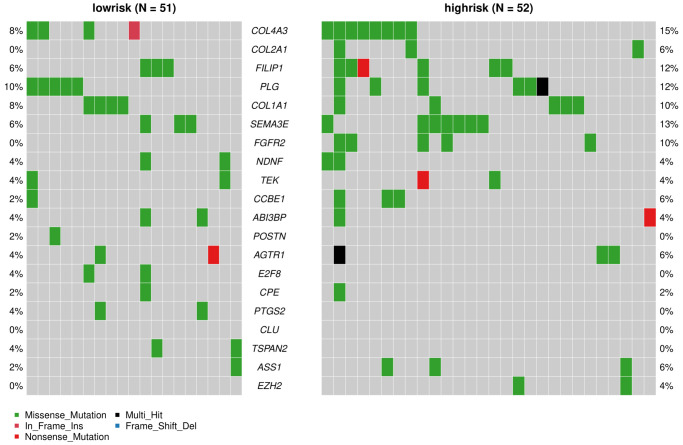
**Analyses of somatic mutation profiles in melanoma samples.** Waterfall plot of detailed mutation information of the top 20 genes in the high- and low-risk groups, with various color annotations to distinguish different mutation types.

**Figure 14:**
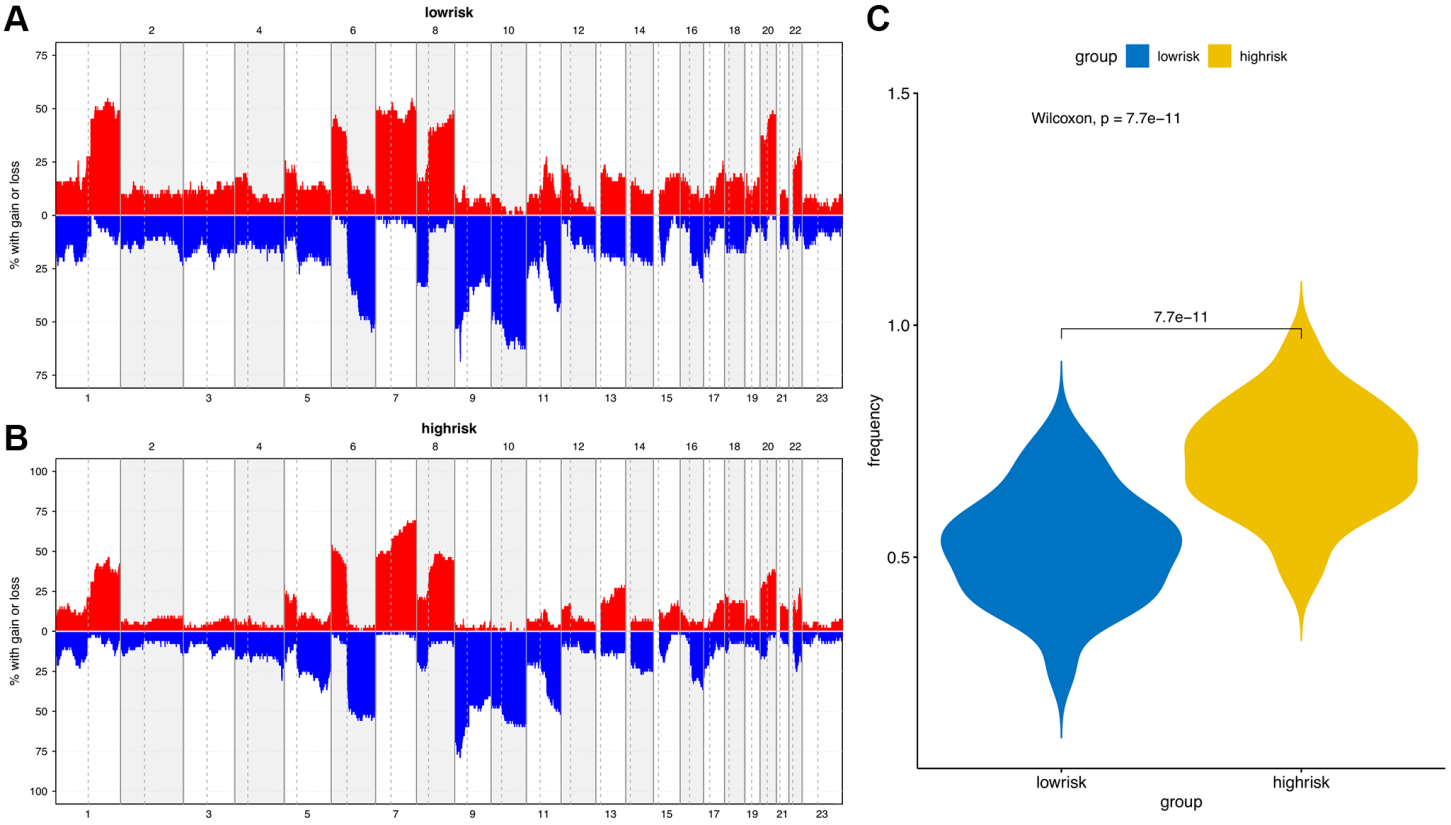
**Copy number variation (CNV) analysis.** (**A**) CNV distribution of low-risk group. (**B**) CNV distribution of high-risk group. (**C**) CNV frequency comparison of different risk groups.

The “maftools” package was utilized to visualize the differences in the mutation patterns of the AFs between the high- and low-risk groups. The mutation information of each AF in individual sample was compared and present in a waterfall plot (Figure 13). The high-risk group had a higher missense mutation frequency. We further evaluated the difference in CNVs between the high-risk group and the low-risk group. As shown in Figure 14, the high-risk group had more obvious levels of amplification and deletion than the low-risk group.

## DISCUSSION

Pathological angiogenesis is a hallmark of cancer, targeting of the AFs have become a promising therapeutic strategy for melanoma. The reported therapeutic targets, such as integrins, vascular endothelial growth factor receptor (*VEGFR1-3*), fibroblast growth factor receptor (*FGFR1-4*), platelet-derived growth factor receptor α (*PDGFRα*), stem cell factor receptor (*KIT*), angiopoietin-2 (*ANGPT2*), E-selectin, the transcription factor Yin Yang 1 (*YY1*) and invasive endothelial cells (ECS) [16–20], can be inhibited by drugs like lenvatinib and propranolol, to delay tumor angiogenesis [17, 21]. Additionally, many clinical studies are evaluating the therapeutic effect of angiogenesis inhibitors for patients with metastatic melanoma [22]. Although there have been many reports on the use of a single AF as treatment strategy for melanoma, there has been no multigene analysis of angiogenic factors to evaluate the prognosis and survival risk of patients with melanoma. Therefore, in this study, we constructed a prognostic risk model of melanoma based on AFs, evaluated its efficacy, and analyzed the clinical genetic characteristics, immune infiltration levels, mutations and immunotherapy responses to provide a new strategy for the treatment of melanoma.

In this study, we first used the NCBI gene and MSigDB database to search and download angiogenesis-related genes as an AF gene set and finally obtained a total of 473 AFs. Then, we integrated the TCGA and GTEX databases to obtain clinical information data of 60498 transcripts, 470 skin melanoma tissue samples and 737 normal tissue samples. The Limma package was used to compare tumor samples with normal samples to identify the significant DEGs and to obtain 49 DE-AFs, including 26 upregulated and 23 downregulated DE-AFs.

KEGG analyses showed that the DE-AFs are mainly involved in the regulation of cancer pathways, including the PI3K/Akt pathway, focal adhesion, and MAPK signaling pathway, which are related to the occurrence and development of melanoma. PI3K/Akt pathway alterations occur in up to 70% of melanomas, participates in tumor angiogenesis, and plays a role in promoting tumor development and inducing drug resistance in melanoma [23–25]. Focal adhesion formation promotes the invasion and migration of melanoma and is related to the regulation of AKT1^E17K^ in the PI3K/Akt pathway [23, 26]. The PI3K/Akt, focal adhesion and MAPK signaling pathways are involved in the regulation of angiogenesis and vascular permeability [24, 27]. The PI3K/Akt pathway can induce the expression of vascular endothelial growth factor (VEGF), nitric oxide, angiopoietin and other angiogenic factors. Accordingly, many inhibitors targeting the PI3K/Akt/mTOR pathway have been developed, which can reduce the secretion of vascular endothelial growth factors and thus reduce angiogenesis [28].

Next, we performed LASSO regression analysis of the DE-AFs to remove redundant factors, 11 significant gene features were screened, including 3 DE-AFs (SPP1, SHH, CD44) with positive coefficients and 8 DE-AFs (APOE, EZH2, ICAM1, LEF1, PTGS2, S100A1, SERPINE2, TIMP1) with negative coefficients. A KM curve was generated to evaluate the relationship between each selected DE-AF and the survival prognosis of melanoma patients. The results showed that the selected 11 DE-AFs were significantly correlated with survival prognosis. Patients with high expression of these DE-AFs tended to have a poorer prognosis. To further assess the prognostic capability of this model, we verified the performance of the model in the TCGA cohort and GEO validation cohort. The AUCs of the discovery cohort and validation cohort were shown as 0.88 and 0.80, respectively, indicating that this model had high accuracy and excellent predictive capacity.

The analysis of clinical features showed that patients with height <170 cm and weight <80 kg, and those with advanced stage and ulcerated melanoma had higher risk scores. Sex is also an important factor that involving in the occurrence of melanoma, but no significant difference was observed. In general, the incidence rate of melanoma in males is higher than that in females, but it is also influenced by other factors, such as location and age [29]. Height is positively correlated with the risk for melanoma, but there is insufficient evidence for the correlation between high body mass index (BMI) and melanoma [30]. In contrast, in male patients with metastatic melanoma who received targeted or immunotherapy, those who were obese had higher progression-free survival and overall survival rates than those with a normal BMI [31]. Pia Vihinen et al. [32] showed that among patients with melanoma, the concentration of angiopoietin in men was significantly higher than that in women. Melanoma secretes a variety of angiogenic factors to promote neovascularization, which is an important factor leading to the ulceration of melanoma and is related to the transformation from the radial growth phase to the vertical growth phase and the metastasis phase [33, 34]. In addition, microvascular density (MVD) is significantly related to melanoma stage [35].

We also collected other genetic characteristics of the patients, including TMB, HRD, tumor neoantigen load and chromosomal instability. Except for the negative correlation of SCNV gene proportion with the risk score, the rest of the genetic characteristics were positively correlated with the risk score. TMB reflects the number of mutations in tumors, and its increase may come from exposure to ultraviolet radiation (UVR), cigarette smoke and other factors [36, 37]. UVR mutagenesis causes nearly all cutaneous melanomas, which correlates with melanoma development and tumor progression [38–40]. Higher TMB can lead to more neo-antigens, increasing the opportunity of T cell recognition, so it can be used in clinical immunotherapy [41]. The highest levels of TMB are known to occur in melanoma and other skin cancers [36]. Higher TMB was associated with worse survival in patients with melanoma and many cancer types [36, 42]. In contrast, higher TMB was associated with longer survival for immune checkpoint inhibitors-treated patients [42]. Hence, checkpoint blocking can reactivate immune recognition and is effective in the treatment of melanoma [36, 43]. The neoantigen load is also an important indicator of checkpoint blocking, which is of great significance for cancer immunotherapy [44]. Moreover, the specific expression of new tumor antigens in the tumor tissue and the specific immune response of new tumor antigens are not readily affected by the complex immune tolerance mechanism, so they are an ideal target for immunotherapy [45]. The high mutation rate in melanoma leads to the expression of a large number of new antigens, which can be effectively targeted by immunotherapy [46]. The production of new antigens involves nonsynonymous genetic changes, including single nucleotide variations (SNVs), insertions and deletions (indels), and gene fusions. We analyzed the relationship between SNV and deletion of indel neoantigens and the risk score and showed that they were positively correlated. Melanoma patients who respond to immunotherapy have decreased mutation and neoantigen loads compared to those at baseline [47]. Extensive chromosomal instability exists in melanoma cells [48]. Hence, we analyzed the relationship between the risk score and the NtAI, LOH, and LSTM scores and found that there was a positive correlation. Somatic copy number alterations (SCNAs) are a recurrent characteristic of malignant cancers and have been identified as drug resistance factors [49]. EPHA3 and FRS2 are SCNA-affected genes whose products participate in angiogenesis and migration and have the potential to be therapeutic targets for melanoma [44, 49].

Immunocyte infiltration analysis showed that memory B cells, macrophages and monocytes were more infiltrated in high-risk patients. B cells and macrophages M2 promote tumor growth and metastasis [50, 51]. Tumor infiltrating B cells can contribute to anti-tumor immune response in melanoma, and the absence of B cells is associated with a poor response to immune checkpoint inhibitors [52, 53]. ANGPT2 can induce therapeutic resistance by increasing angiogenesis and immunosuppressive activity in the tumor microenvironment. These results indicate that antiangiogenic drugs and immune checkpoint blockers can be combined to treat melanoma in the future. In addition, the tumor microenvironment (TME) immune cell/stromal cell ratio is also an important indicator of tumor development [15]. The immune score and tumor purity score were positively correlated with the survival rate [54], and the higher the immune score was, the better the prognosis of the patients [55]. We used ESTIMATE to evaluate the differences in immune score, stromal score and tumor purity between high-risk and low-risk patients. The results showed that low-risk patients had higher immune scores and tumor purity, indicating that AFs have the same prognostic value as the immune score. The results of the TIDE online analysis tool also showed that low-risk patients had better immunotherapy responses, which may be related to the immune score.

We also analyzed the difference in AF factor mutation levels and CNVs among high- and low-risk patients. The results showed that the gene mutation rate of AFs in the high-risk group was higher than that of the low-risk group, and missense mutations accounted for the majority of the mutations. The CNV amplification level, deletion level and variation frequency were more obvious in the high-risk group than in the low-risk group. The most obvious difference in the missense mutation rate between the two groups was in FGFR2, which was mutated more frequently in the high-risk group (10%) than in the low-risk group (0%). FGFR2 missense mutation also occurs in endometrial cancer, diffuse gastric cancer and triple-negative breast cancer. FGFR inhibitors, as tumor-targeted therapy drugs, have shown significant effects in preclinical model experiments [56, 57]. Copy number variation (CNV) is an important factor in structural variation (SV), which is closely related to the progression of melanoma [58, 59]. The correlation between angiogenesis and CNVs in single or multiple genes has been observed in breast cancer, gastric cancer and other tumors [60, 61], but it has not been previously reported in melanoma.

In summary, this study integrated multiomics approaches, including transcriptome analysis to identify DE-AFs, genome analysis to identify risk-related variants, copy number analysis to identify the distribution of amplification and deletion variants in different risk groups, and systematically analyzed the molecular mechanisms related to cutaneous melanoma. We also combined univariate, multivariate and machine learning algorithms to screen prognosis-related AFs, further compared and verified them in two independent datasets from the TCGA and GEO databases. Finally, we proved that the AFs screened in this study have high-risk prediction efficiency. The limitation of this study is that all of the analyses are based on the confirmed AFs recorded in the databases, so other unknown angiogenic factors were not evaluated. We expect potential angiogenic factors or skin melanoma related AF-genes be included in future studies, which therefore could further improve the present prediction model.

## MATERIALS AND METHODS

### Data acquisition

The gene expression profiles and corresponding clinical information of patients with melanoma were obtained from the TCGA (https://portal.gdc.cancer.gov/) and GTEx (https://www.gtexportal.org/home/multiGene QueryPage) databases,

### Extraction of differentially expressed AFs

A total of 473 genes were obtained from the NCBI gene (https://www.ncbi.nlm.nih.gov/gene/) and MSigDB (http://www.gsea-msigdb.org/gsea/msigdb/index.jsp) databases and identified as AF gene sets. Differentially expressed genes (DEGs) between tumor and normal tissues were identified using the R package “limma”. Absolute log2-fold-change >95% confidence intervals (CIs) and *P* < 0.05 were considered statistically significant to screen out upregulated and downregulated DEGs. The overlapping gene set between AFs and DEGs was referred to as differentially expressed AFs (DE-AFs). A volcano map was used to show the distribution of upregulated and downregulated DEGs. The samples were divided into two groups based on the DE-AFs were analyzed and visualized by hierarchical clustering.

### Pathway enrichment analysis

Kyoto Encyclopedia of Genes and Genomes (KEGG) analyses were carried out by using the “Cluster Profiler package” in R for functional annotation and pathway enrichment. *P* < 0.05 was set as the cutoff point to screen the significant functions of DE-AFs. The top ten noteworthy functions were visualized by using the “ggplot package” in R.

### Construction and validation of prognostic signatures of DE-AFs

Univariate Cox regression analysis was performed to screen out the significant overall survival (OS)-related AFs from all DE-AFs. Kaplan–Meier (KM) curves were drawn to evaluate the relationship between each AF and the survival and prognosis of melanoma patients. The DE-AFs of survival-related modules were integrated into least absolute shrinkage and selection operator (LASSO) regression analysis for the identification of prognostic risk signatures. The risk score of the DE-AF signature for each sample was calculated as follows:


$$\lambda_{t}=\beta_{1}X_{1}+\beta_{2}X_{2}+\;\cdots \;+\beta_{k}X_{k}$$

where *k* is the number of selected AFs, *X_k_* is the expression value of gene *k*, *β_k_* is the coefficient of gene *k*, and *λ_t_* is the risk score of sample *t*. All patients were divided into a high-risk group and a low-risk group according to the median risk score.

Finally, KM analysis was conducted to evaluate the differences in OS rates between the high- and low-risk groups in the TCGA and GEO cohorts. The area under the curve (AUC) of the ROC curve was calculated to examine the DE-AF signature performance using the time ROC R package.

### Difference analysis of the AF risk score, clinical features and genetic characteristics

We collected clinical indicators, including sex, height, weight, melanoma ulceration, treatment, and stage, and compared the differences in survival indicators in different risk groups. A box plot was used to visualize the comparative analysis, Wilcoxon test was used to calculate the significance *P* value. *P* < 0.05 was used as the cutoff to screen out significant clinical features. To explore the differences in genetic characteristics, we analyzed the relationship between the risk score and mutation load, neoantigen load and chromosomal instability. Pearson’s correlation analysis was used to calculate the risk score and the correlation coefficient between different genetic characteristics.

### Comparison of the immune landscape between the high and low risk score groups

To explore the differences in immune cell subtypes, the CIBERSORT algorithm was used to quantify the infiltration degree of 22 types of immune cells in each patient, and the Wilcoxon test was used to calculate the difference in the level of each kind of immune cell in samples with different risk scores. A *t*-test was used to calculate the *P* value in the differential analysis. *P*-value <0.05 was considered as the cutoff value.

Stromal cells and immune cells are the main normal cells present in tumor tissues and participate in tumor development. We used the ESTIMATE database (https://bioinformatics.mdanderson.org/estimate/) to estimate the immune score, matrix score and tumor purity of each sample and compared differences in the three indicators between the different risk groups. Then, the potential response to PD-1/CTLA4 immunotherapy for each sample was predicted using the TIDE algorithm (http://tide.dfci.harvard.edu/).

### Comparisons of mutation patterns in AFs and CNVs between high and low-risk score groups

We used maftools to download maf format mutation spectrum data from the TCGA and visualized an oncoplot to show the different AF mutations in different risk groups. Then, copy number variation (CNV) data were downloaded from the TCGA to compare the level of amplification and deletion in different risk groups.

## Supplementary Materials

Supplementary Figure 1

Supplementary Table 1

Supplementary Table 2

Supplementary Table 3

Supplementary Table 4
