# Supplementary material for: Construction and validation of a prognostic risk model of angiogenesis factors in skin cutaneous melanoma
Source: Aging (Albany NY). 2022 Feb 14;14(3):1529–48. doi: 10.18632/aging.203895 (PMC8876895; doi:10.18632/aging.203895)
Supplement: Supplementary Figure 1 [file aging-14-203895-s001.pdf]

SUPPLEMENTARY FIGURE

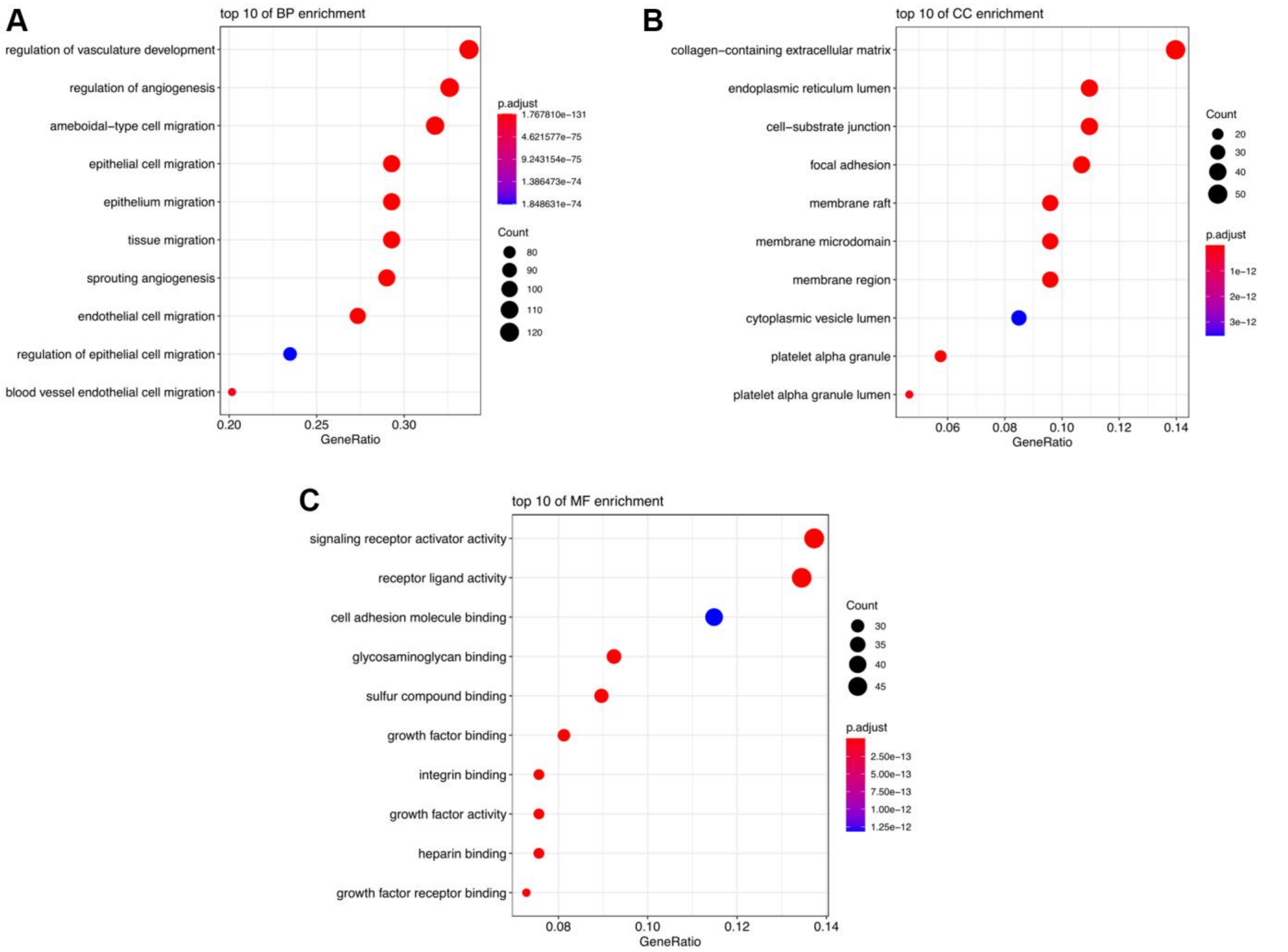

Supplementary Figure 1. Functional annotation analysis of DE-AFs. Gene Ontology (GO) enrichment analysis results show the top 10 terms (**A**) biological process (BP) enrichment, (**B**) cellular component (CC) enrichment and (**C**) molecular function enrichment (MF) enrichment.
