# Supplementary material for: Construction and validation of a prognostic risk model of angiogenesis factors in skin cutaneous melanoma
Source: Aging (Albany NY). 2022 Feb 14;14(3):1529–48. doi: 10.18632/aging.203895 (PMC8876895; doi:10.18632/aging.203895)
Supplement: Supplementary Table 4 [file aging-14-203895-s005.pdf]

SUPPLEMENTARY TABLE

Supplementary Table 4. The DE-AFs list of melanoma.

AF	logFC	AveExpr	*t*	*P*.Value	adj.*P*.Val	B	color	mean_normal	mean_tumor
FGFR2	−7.19547	8.851778	−70.3426	0	0	974.5971	downregulate	11.65	4.455
ASS1	−5.47164	11.07928	−52.0117	1.5650907884101e-310	5.26E-308	701.3007	downregulate	13.207	7.736
CLU	−5.02769	13.58689	−43.1848	4.04E-247	4.16E-245	555.4083	downregulate	15.542	10.514
AGTR1	−5.15804	6.453298	−40.9502	1.48E-230	1.12E-228	517.2963	downregulate	8.459	3.301
EDN1	−4.03543	7.797869	−40.0595	6.57E-224	4.23E-222	502.0045	downregulate	9.367	5.332
TSPAN2	−4.12576	7.208263	−38.9262	2.01E-215	1.07E-213	482.4792	downregulate	8.813	4.687
NDNF	−6.00331	6.652656	−37.8029	5.53E-207	2.52E-205	463.0621	downregulate	8.987	2.984
TEK	−3.32996	9.162279	−37.3781	8.71E-204	3.64E-202	455.7059	downregulate	10.457	7.127
CCBE1	−4.27214	6.4274	−35.0745	2.07E-186	6.42E-185	415.7284	downregulate	8.089	3.817
TDGF1	−4.61346	3.90641	−34.9212	2.98E-185	9.03E-184	413.0651	downregulate	5.701	1.087
TACSTD2	−6.24091	9.609409	−34.683	1.87E-183	5.46E-182	408.9273	downregulate	12.036	5.796
RSPO3	−3.57349	6.942257	−33.5873	3.51E-175	8.59E-174	389.8946	downregulate	8.332	4.758
CPE	−3.10963	11.00203	−32.9837	1.26E-170	2.87E-169	379.4174	downregulate	12.211	9.102
FILIP1	−2.96884	7.901768	−31.6461	1.49E-160	2.89E-159	356.242	downregulate	9.056	6.087
COL4A3	−4.73086	6.563405	−29.8892	2.21E-147	3.52E-146	325.9446	downregulate	8.403	3.672
CNN1	−4.47938	9.436576	−28.0722	6.99E-134	9.03E-133	294.8925	downregulate	11.179	6.699
ABI3BP	−3.19852	9.987383	−27.8354	3.90E-132	4.90E-131	290.8742	downregulate	11.231	8.033
SHH	−4.80071	4.39764	−26.7234	5.65E-124	6.21E-123	272.1057	downregulate	6.265	1.464
PTGS2	−3.30937	8.330939	−24.6762	3.46E-109	3.03E-108	238.1021	downregulate	9.618	6.309
FABP4	−4.37392	7.961728	−22.2206	5.54E-92	3.81E-91	198.5512	downregulate	9.663	5.289
CXCL6	−3.299	3.982018	−22.0959	3.97E-91	2.69E-90	196.5859	downregulate	5.265	1.966
PLG	−4.68062	4.319591	−20.6137	3.94E-81	2.28E-80	173.6117	downregulate	6.14	1.459
SEMA3E	−2.97677	6.254211	−17.6636	2.77E-62	1.17E-61	130.3213	downregulate	7.412	4.435
SERPINE2	5.690374	12.45138	67.38687	0	0	933.3444	upregulate	10.238	15.929
LEF1	3.934312	9.524067	40.9369	1.86E-230	1.40E-228	517.068	upregulate	7.994	11.928
E2F7	4.003252	6.149316	39.09211	1.15E-216	6.21E-215	485.342	upregulate	4.592	8.596
CCNE1	2.531535	7.078473	37.17611	2.90E-202	1.18E-200	452.2053	upregulate	6.094	8.626
E2F3	1.596493	9.858777	35.18054	3.28E-187	1.02E-185	417.5702	upregulate	9.238	10.834
EZH2	2.537597	8.371034	33.35314	2.05E-173	4.87E-172	385.8295	upregulate	7.384	9.922
S100A1	4.887598	10.54034	32.22082	7.06E-165	1.46E-163	366.1912	upregulate	8.64	13.527
STXBP1	2.308574	11.73167	29.85897	3.71E-147	5.87E-146	325.426	upregulate	10.834	13.142
CKS2	1.792367	9.229999	25.45077	9.66E-115	9.20E-114	250.8731	upregulate	8.533	10.325
E2F2	3.093279	6.869216	24.87974	1.22E-110	1.09E-109	241.4458	upregulate	5.666	8.76
TIMP2	1.668293	14.55965	24.50072	6.17E-108	5.33E-107	235.2264	upregulate	13.911	15.579
HEY1	2.056313	9.269506	23.69149	3.29E-102	2.60E-101	222.0575	upregulate	8.47	10.526
TIMP1	1.835048	13.25034	22.55938	2.58E-94	1.83E-93	203.9118	upregulate	12.537	14.372
CD44	2.104637	13.97978	21.69526	2.14E-88	1.38E-87	190.3058	upregulate	13.161	15.266
MMP9	3.152379	9.03923	21.56438	1.65E-87	1.06E-86	188.2651	upregulate	7.813	10.966

APOE	2.922432	13.85134	21.52445	3.08E-87	1.96E-86	187.6436	upregulate	12.715	15.637
E2F8	2.433198	5.809441	19.39719	3.56E-73	1.81E-72	155.3313	upregulate	4.863	7.296
SCG2	2.654919	6.558604	18.04244	1.29E-64	5.68E-64	135.6741	upregulate	5.526	8.181
MCAM	1.729986	12.9065	17.83499	2.46E-63	1.06E-62	132.7351	upregulate	12.234	13.964
SPHK1	1.679456	8.901242	17.47058	4.15E-61	1.73E-60	127.6206	upregulate	8.248	9.928
PGF	1.636613	9.059629	16.22925	9.92E-54	3.69E-53	110.6847	upregulate	8.423	10.06
COL1A1	1.902429	14.34453	15.7813	3.76E-51	1.34E-50	104.7684	upregulate	13.605	15.507
SPP1	2.686247	11.72929	15.70787	9.85E-51	3.48E-50	103.8088	upregulate	10.685	13.371
ICAM1	1.580152	12.18022	14.95105	1.69E-46	5.57E-46	94.09498	upregulate	11.566	13.146
POSTN	1.831015	11.40336	12.99393	3.15E-36	8.59E-36	70.56077	upregulate	10.691	12.522
COL2A1	1.764182	4.215848	9.545315	7.23E-21	1.46E-20	35.451	upregulate	3.53	5.294
